# Computed Tomography Planning for Transcatheter Mitral Valve Replacement

**DOI:** 10.1016/j.shj.2022.100012

**Published:** 2022-04-26

**Authors:** Go Hashimoto, Bernardo B.C. Lopes, Hirotomo Sato, Miho Fukui, Santiago Garcia, Mario Gössl, Maurice Enriquez-Sarano, Paul Sorajja, Vinayak N. Bapat, John Lesser, João L. Cavalcante

**Affiliations:** a Cardiovascular Imaging Research Center and Core Lab, Minneapolis Heart Institute Foundation, Minneapolis, Minnesota, USA; b Valve Science Center, Minneapolis Heart Institute Foundation, Minneapolis, Minnesota, USA; c Minneapolis Heart Institute, Abbott Northwestern Hospital, Minneapolis, Minnesota, USA

**Keywords:** MDCT, Mitral regurgitation, TMVR

## Abstract

Transcatheter mitral valve replacement (TMVR) is a rapidly evolving treatment for mitral regurgitation. As with transcatheter aortic valve replacement, multidetector computed tomography analysis plays a central role in defining the candidacy, device selection and safety for TMVR procedures. This contemporary review will describe in detail the multidetector computed tomography data collection, analysis, and planning for TMVR procedures in patients with native mitral regurgitation as well as in those with failed surgical prosthetic mitral valve replacement or surgical mitral valve repair.

## Introduction

Mitral regurgitation (MR) occurs from different mechanisms involving the mitral valve (MV) complex. MR is broadly classified as degenerative MR when caused by primary leaflet disorders (fibroelastic deficiency, MV prolapse, rheumatic disease, endocarditis, etc.) and as functional MR (FMR), which can be caused by left atrial and/or left ventricular remodeling. Although both MR categories are potentially suitable for transcatheter edge-to-edge MV repair (TEER), patient-specific anatomical features may preclude the use of this therapy.

Transcatheter mitral valve replacement (TMVR), on the other hand, can be broadly divided as for the treatment of native MR or for the treatment of patients with failure of prior surgical MV replacement or MV repair. TMVR for native MR is indicated for high-risk surgical candidates and is currently under investigational research studies in the United States of America. TMVR devices for native MV replacement include Tendyne (Abbott Structural, Santa Clara, California), Intrepid (Medtronic, Inc), SAPIEN M3, EVOQUE (Edwards Lifesciences, Irvine, California), and Cephea (Cephea Valve Technologies, San Jose, California). TMVR has also been approved by the U.S. Food & Drug Administration (FDA) for the treatment of failed bioprosthetic valves (valve-in-valve [ViV] TMVR) and of prior MV annuloplasty repair (valve-in-ring [ViR] TMVR) with the use of a balloon-expandable transcatheter aortic valve device (Edwards SAPIEN 3, Edwards Lifesciences). In patients not suitable for TEER, given the presence of mitral annular calcification (MAC) and small MV area, the option for TMVR valve-in-MAC (ViMAC) has also been performed under research registries either with specific native TMVR devices or with SAPIEN 3.

Multimodality imaging is key for successfully selecting patients suitable for TMVR. Both echocardiography and multidetector computed tomography (MDCT) are necessary imaging tools for the MR diagnosis, evaluation of severity and mechanism, TMVR procedure planning, and follow-up of patients after TMVR. This review presents comprehensive updated information on MDCT analysis for TMVR screening, procedure planning, and patient follow-up of patients.

## MV Anatomy for TMVR Planning

### Mitral Leaflets

**Figure 1:**
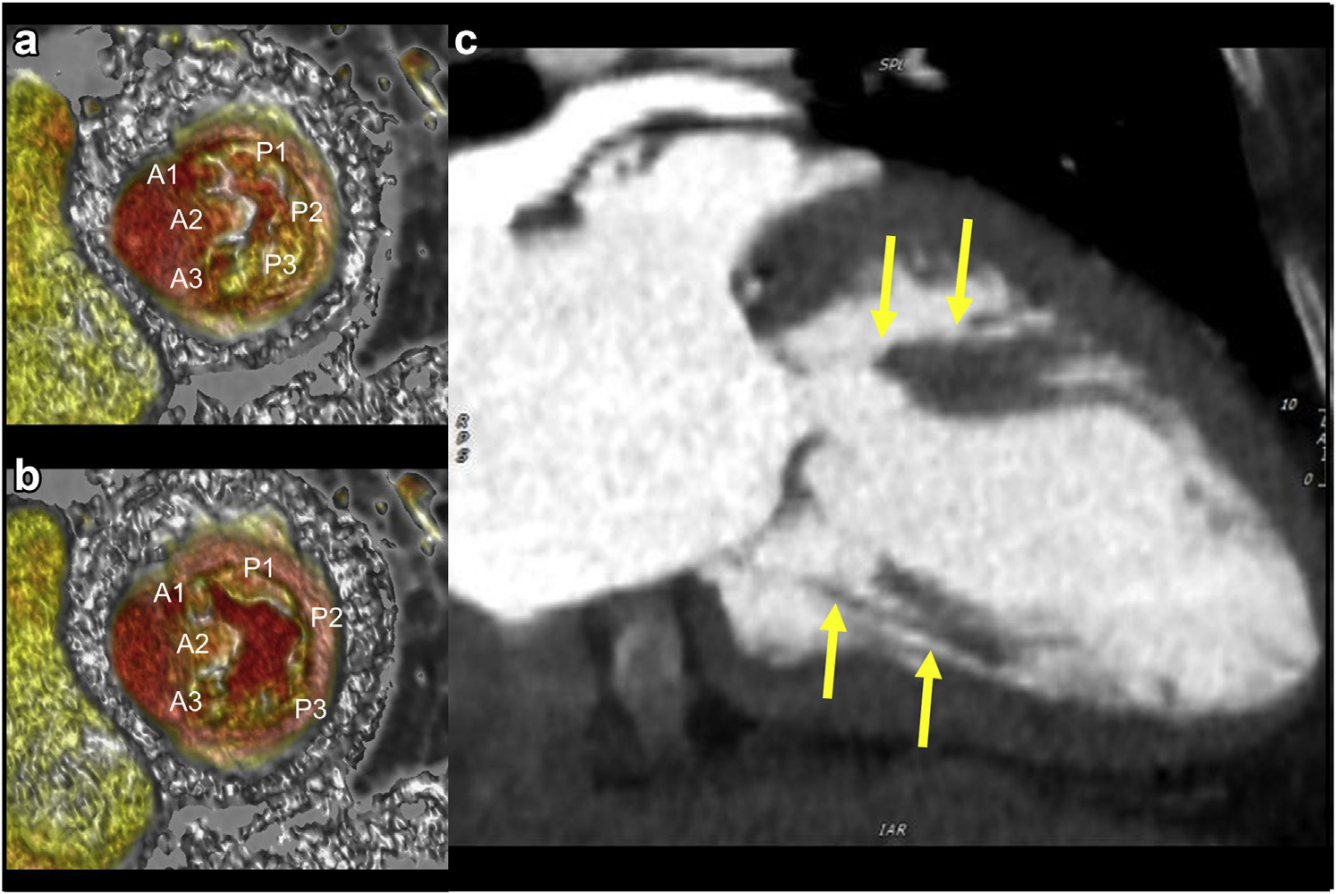
**Anatomy of the mitral valve and subvalvular apparatus.** (a) Anterior mitral leaflet (from lateral to medial, A1, A2, and A3) and posterior mitral leaflet (P1, P2, and P3) in end-systolic cardiac phase. (b) Anterior mitral leaflet (from lateral to medial, A1, A2, and A3) and posterior mitral leaflet (P1, P2, and P3) in end-diastolic cardiac phase. (c) Yellow arrows: anterolateral and posteromedial papillary muscles with chordae tendineae in long-axis 2-chamber view.

The anterior MV leaflet (AML) and posterior MV leaflet are structurally and functionally different.^1^ They connect to the chordae tendineae and papillary muscles attached to the fibrous mitral annulus at the atrioventricular junction. The AML is larger and covers approximately two-thirds of the mitral annular opening. It facilitates the vorticial flow into the posterior wall of the left ventricle, preparing it for ejection at the left ventricular outflow tract (LVOT). The posterior leaflet is shorter in length and divided into 3 scallops by 2 indentations. Deeper indentations on the anterolateral side and the posteromedial side are called commissures separating the 2 ​MV leaflets^2^ (Figure 1).

In the context of TMVR, the MV leaflets may serve as a fixation for TMVR devices (in which the device grabs the leaflets) but may also contribute to LVOT obstruction (LVOTO) after device implant (e.g., after ViR or native TMVR). Therefore, measurement of the AML length and the location of papillary muscle insertion into the leaflets should be assessed, identifying any potential interference with device fixation.^3^ Typically, an elongated AML (i.e., >25 ​mm in length and therefore larger than the LVOT diameter) seems to pose some relative risk for LVOTO but is not the sole determinant.

### Mitral Annulus

The mitral annulus anatomically is a nonplanar 3-dimensional saddle-shaped structure that shares close proximity to the aortic valve via the mitral-aortic intervalvular fibrosa. Mitral annulus sizing is an important step in TMVR planning. Mitral annulus dimensions are dynamic and therefore should be measured in both systolic and diastolic phases. MDCT scan data acquisition, therefore, needs to be not only electrocardiogram (ECG) gated but also must encompass the entire cardiac cycle, preferably without dose modulation. Changes in the mitral annulus size in degenerative MR are more dynamic than in functional MR.^4^ Most TMVR devices require some degree of oversizing in multiple parameters (mitral annulus perimeter, mitral annulus area, intercommisural or anteroposterior diameter) to avoid paravalvular leak (PVL) and/or device embolization.

**Figure 2:**
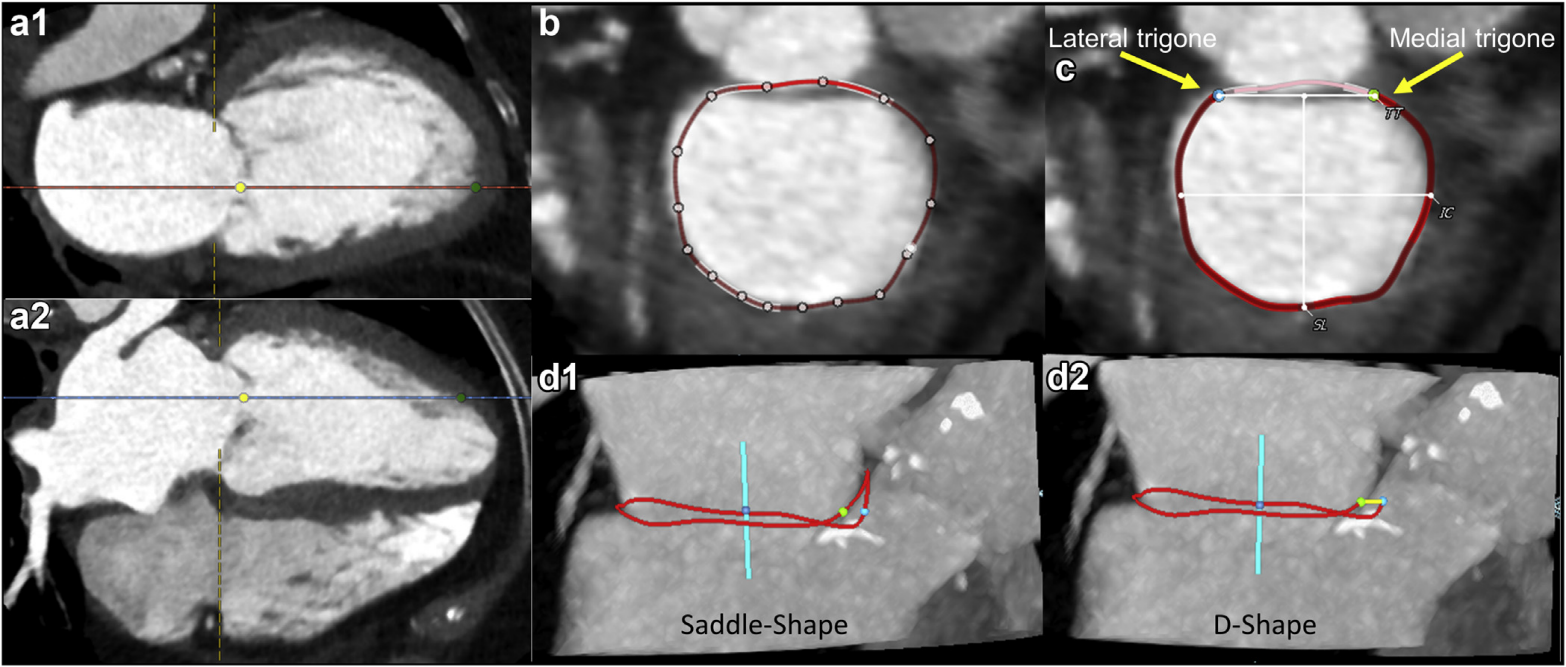
**TMVR analysis of the mitral annulus by MDCT.** (a1 and a2) Determine the center of the mitral valve (yellow dot) and LV apex (green dot) in 2 orthogonal views. This defines an MV centroid axis, which will be used for the next step, which is the identification of the mitral annulus. (b) Interpolated cubic spline is created using a semi-automated way rotating around the mitral annulus centroid point created on the prior step. Sixteen seed points are deposited at the base of the MV leaflets and connected to create the mitral annulus. Each point can be selected and position corrected. The area of the mitral-aortic curtain always appears thicker delineating the transition to a less fibrotic annulus. (c) Define the lateral and septal trigones recognized by the transition point whereby aortic root disappears from the orthogonal plane. (d1) Saddle-shaped mitral annulus. (d2) D-shaped mitral annulus.

For TMVR screening and planning, the mitral annulus segmentation is typically performed using dedicated software with the semi-automated creation of multiplanar reformat images and a user-defined interpolated cubic spline connecting 16 seed points deposited at the base of the MV leaflets. The 2 mitral annular trigones are defined by the MV commissures at the transition between the AML and posterior MV leaflet. Two geometrical shapes for the native MV annulus can be considered: (1) a saddle-shaped, which incorporates the mitral-aortic intervalvular fibrosa; or (2) a D-shaped mitral annulus, which truncates the anterior horn into a straight line connecting the 2 trigones.^5^ Figure 2 demonstrates the mitral annulus measurements using dedicated software.

MAC often occurs in elderly patients with concomitant aortic stenosis and/or with chronic renal failure. This higher risk population represents a challenging cohort that was initially excluded from TMVR trials. However, increased experience with both balloon-expandable SAPIEN 3 transcatheter aortic valve replacement device and dedicated Tendyne (Abbott Structural, Menlo Park, California) TMVR device have been reported with favorable results, provided no LVOTO is identified on screening analysis.^6^ Further expansion of TMVR indication into this population will require continued iterations in device design and a deeper understanding of the interaction of TMVR with MAC via post-TMVR implantation MDCT imaging (see the section on TMVR in MAC).

### Identifying Etiology and Mechanism of MR on MDCT

**Figure 3:**
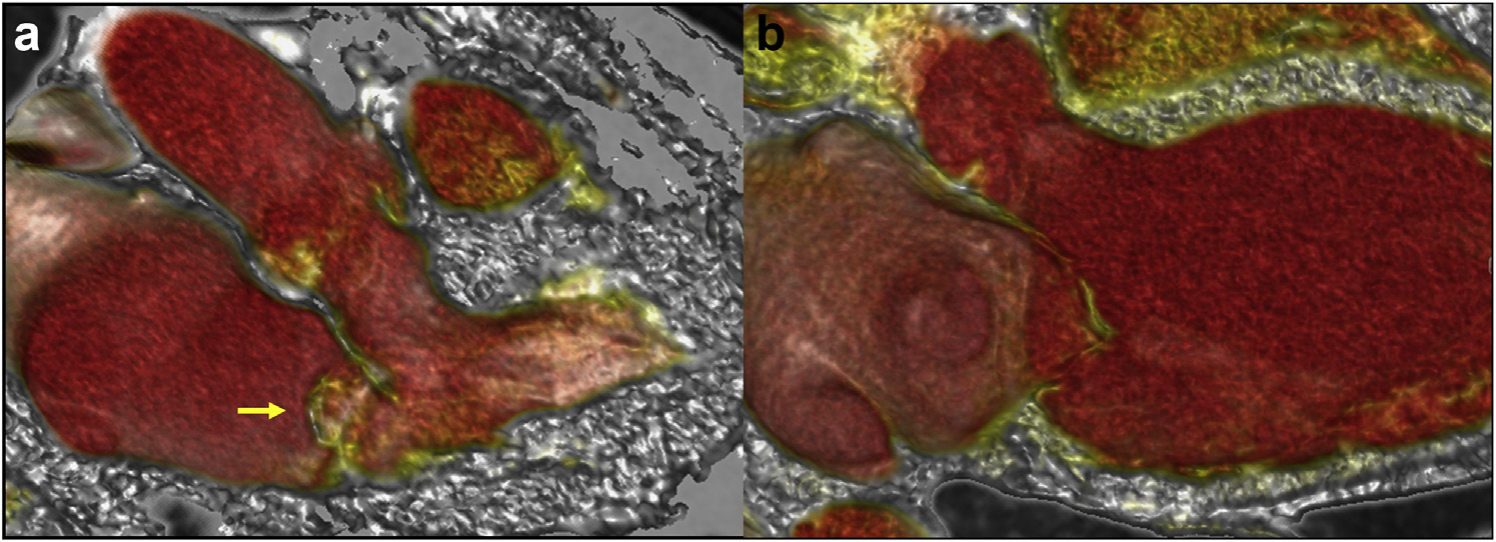
**Primary and secondary mitral regurgitation seen through multiphasic MDCT.** (a) Primary mitral valve regurgitation. Posterior MV leaflet prolapse and a large flail gap (yellow arrow) seen at end-systolic cardiac phase in long-axis 3-chamber view. (b) Secondary mitral valve regurgitation. Both anterior and posterior leaflets are apically tethered along with subvalvular apparatus due to severe dilation and left ventricular remodeling causing restricted MV leaflet motion and incomplete coaptation.

Both transthoracic and transesophageal echocardiography (TEE) are established imaging methods for the diagnosis of MR severity and etiology. Improvements in MDCT technology (particularly dual-source scanner platforms with high temporal resolution) have allowed high-quality functional evaluation, which is very complementary to echocardiography. Functional 3D MDCT can be especially useful when MR etiology is unclear, by combining comprehensive assessment of subvalvular components and detailed quantification of the mitral annulus, left atrial, and left ventricular remodeling (Figure 3).

## TMVR MDCT Data Acquisition and Reconstruction

### MDCT Acquisition Protocol

The MDCT acquisition protocol for TMVR planning will vary depending on scanner type, iodine contrast concentration, patient’s renal function, and body habitus. Accordingly, each institution should customize its acquisition protocol for TMVR. For a comprehensive review of this topic, which is beyond our scope, see a study by Pulerwitz et al.^7^

At our center, we largely use the third generation MDCT dual-source Somatom Force (Siemens Healthineers, Erlangen, Germany) scanner, which has the best temporal resolution in class (66 msec), allowing for consistent diagnostic imaging in patients with higher heart rates and atrial fibrillation. As such, premedication for heart rate control is typically not used. Volumetric MDCT scanners are recommended, as they provide whole heart coverage (e.g., ≥14 cm in the z-axis coverage) at a single heartbeat, albeit with slower temporal resolution than dual-source scanners.

**Figure 4:**
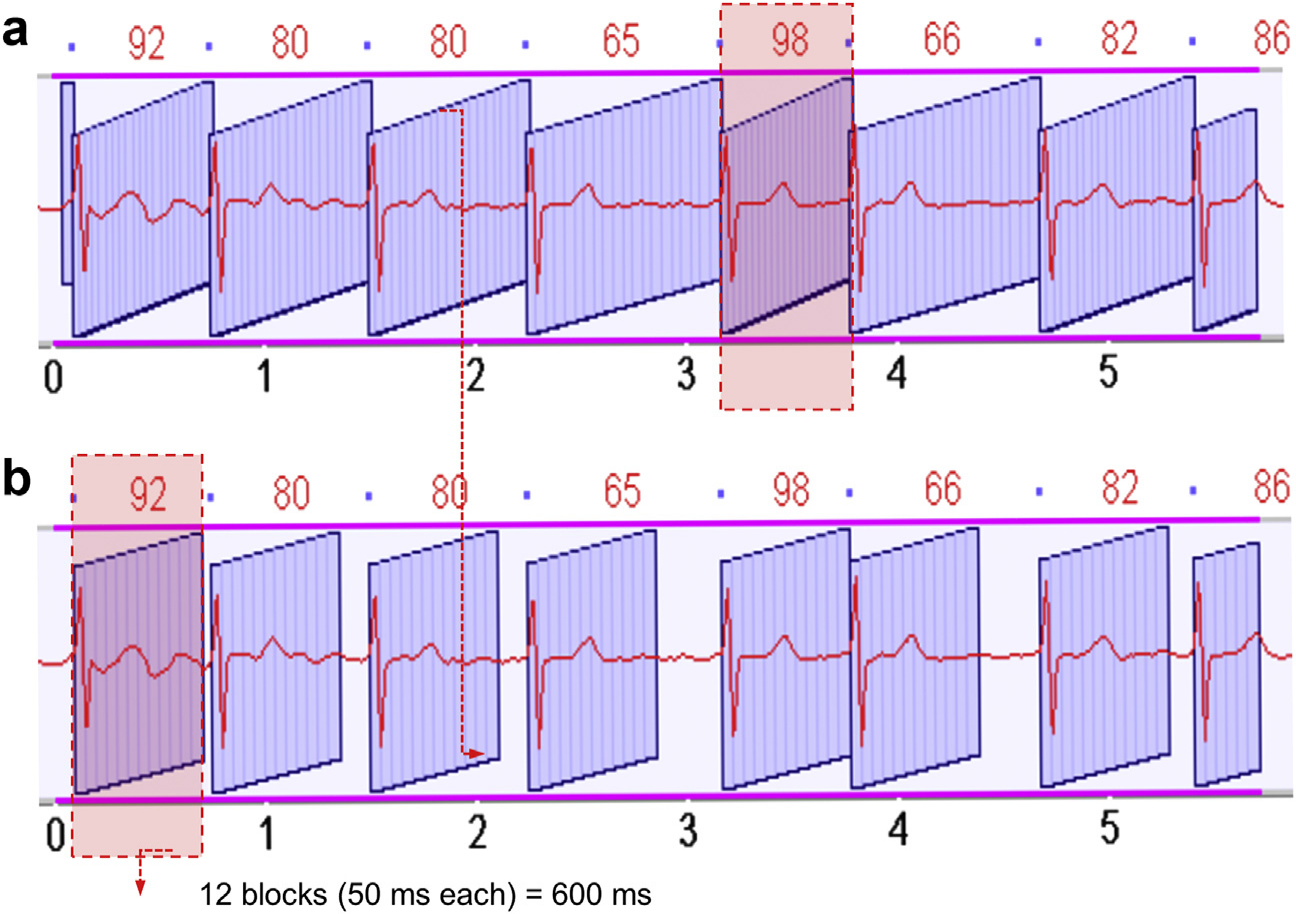
**Absolute milliseconds multiphasic MDCT reconstruction for irregular heart rhythm.** The shortest R-R interval (fastest heart rate) is identified; in this case, 98 beats per minute (panel a, red dashed box). Divide 60,000 ms by the shortest heart rate (60,000/98 = 612 ms). Then round it to the closest 50 ms increment (i.e., 600 ms) and make a functional reconstruction from 0 to 600 ms at 50 ms increments (panel b).

The acquisition protocol starts with a single contrast bolus (iodine concentration of 350 mg/mL and total volume of 50-100 cc) injection with a flow rate of 4-7 mL/s, followed by 60 mL of a 100% saline chaser. Then, the cardiac scan acquisition is triggered using a bolus tracking method with the ROI at the descending aorta at the carina level after an attenuation threshold that varies according to the kV selected (80-120 kVp, according to the patient’s body mass index), typically at 120 HU. A retrospective ECG gated scan (full cardiac cycle coverage) is performed without dose modulation. After the cardiac scan, a high-pitch spiral scan immediately follows to allow for the assessment of appropriate transapical access planning with full chest imaging. Functional images are reconstructed in isotropic voxels of 1.0 mm at every 5% of R-R interval (0-95%). In cases with significant heart rate variability, manual ECG editing and absolute reconstruction at every 50 milliseconds based on the duration of the shortest R-R interval are performed to reduce motion artifacts, as exemplified in Figure 4. Acquired data sets are transferred and offline analyzed at a workstation with dedicated postprocessing software for TMVR planning.

High-quality functional images are necessary for pre-TMVR MDCT assessment. Although the minimum requirement for TMVR would be a 64-detector-row single-source MDCT scanner, this platform can be challenging and often problematic, given the longer acquisition time and breath-hold need, increasing the likelihood for misregistration and motion artifacts, particularly in symptomatic patients who often have arrhythmias and/or faster heart rates. Suboptimal temporal resolution can further amplify such imaging artifacts and make TMVR planning difficult, particularly in the presence of mitral annular calcifications, high-density prosthetic materials, and cardiac arrhythmias.

## Neo-LVOT Assessment

Screening and planning for TMVR includes assessment of risk of LVOTO, a potentially fatal complication of TMVR. After TMVR device implantation, the device struts displace the native AML toward the interventricular septum, thereby creating a neo-LVOT area confined by the deflected AML, the strut of the TMVR device, and the basal interventricular septum.^8^ Also, if the TMVR device does not have an open cell at the end, such as seen with a balloon-expandable transcatheter heart valve (THV), the protruding ventricular end of the device, along with the AML, may also cause and/or contribute to LVOTO.

**Figure 5:**
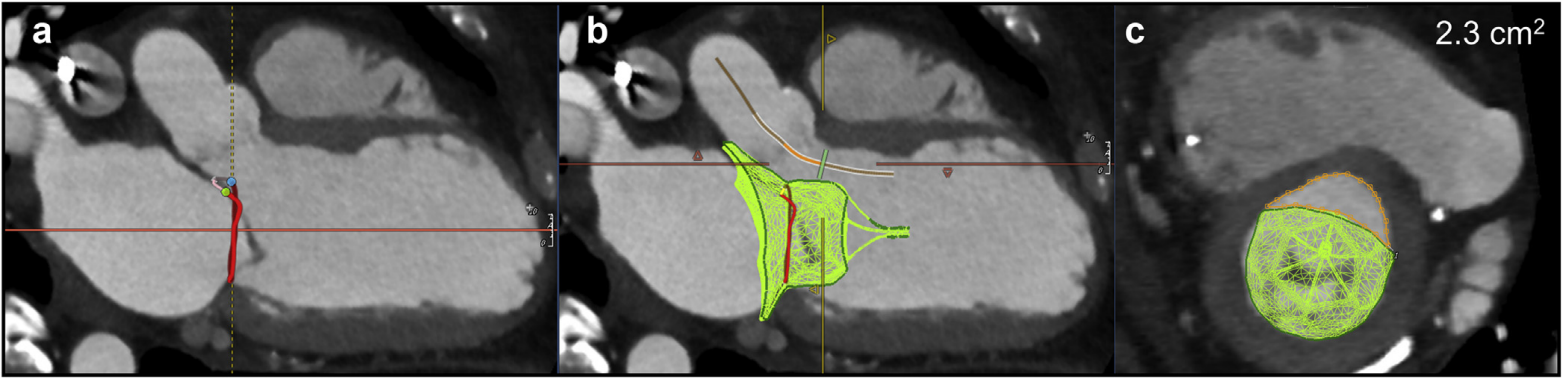
**The methodology for preprocedure Neo-LVOT area assessment using specific TMVR Tendyne Device modeling.** (a) Define mitral annulus plane in the midsystolic cardiac phase. (b) Insert the virtual TMVR device model, which has adequate oversizing relative to mitral annulus, and draw centerline for the Neo-LVOT (solid orange line). (c) Measure Neo-LVOT area at the narrowest point through Neo-LVOT centerline axis. A large Neo-LVOT area is predicted at 2.3 cm^2^ (orange area).

Neo-LVOT area thresholds predictive of LVOTO after TMVR are typically ≤1.7-2.0 cm^2^^9^^,^^10^ and were traditionally measured at the end of systole. We recommend that instead of being more conservative with end-systolic neo-LVOT area measurements, these measurements should be performed at mid-systole when the aortic valve is still open. The midsystolic cardiac phase with less cardiac motion/blurring should be chosen to allow adequate segmentation and analysis. If neo-LVOT area is <2.0 cm^2^, all systolic phases should be evaluated and the mean neo-LVOT area value used to reference LVOTO prediction. This has to do with the fact that most left ventricular (LV) stroke volume is ejected during early to mid-systole, thus using only the end-systolic phase would be overconservative, excluding a significant proportion of patients that could still benefit from TMVR therapies.^11^ Furthermore, these values should not be treated as dichotomous because the midsystolic neo-LVOT area is not the only determinant of the risk of LVOTO, which also includes the LV geometry and contractility, septal thickness, AML length, and aortic-mitral angle. The analysis of native mitral TMVR planning using Tendyne device (Abbott Structural), including the neo-LVOT area measurement, is shown in Figure 5.

**Figure 6:**
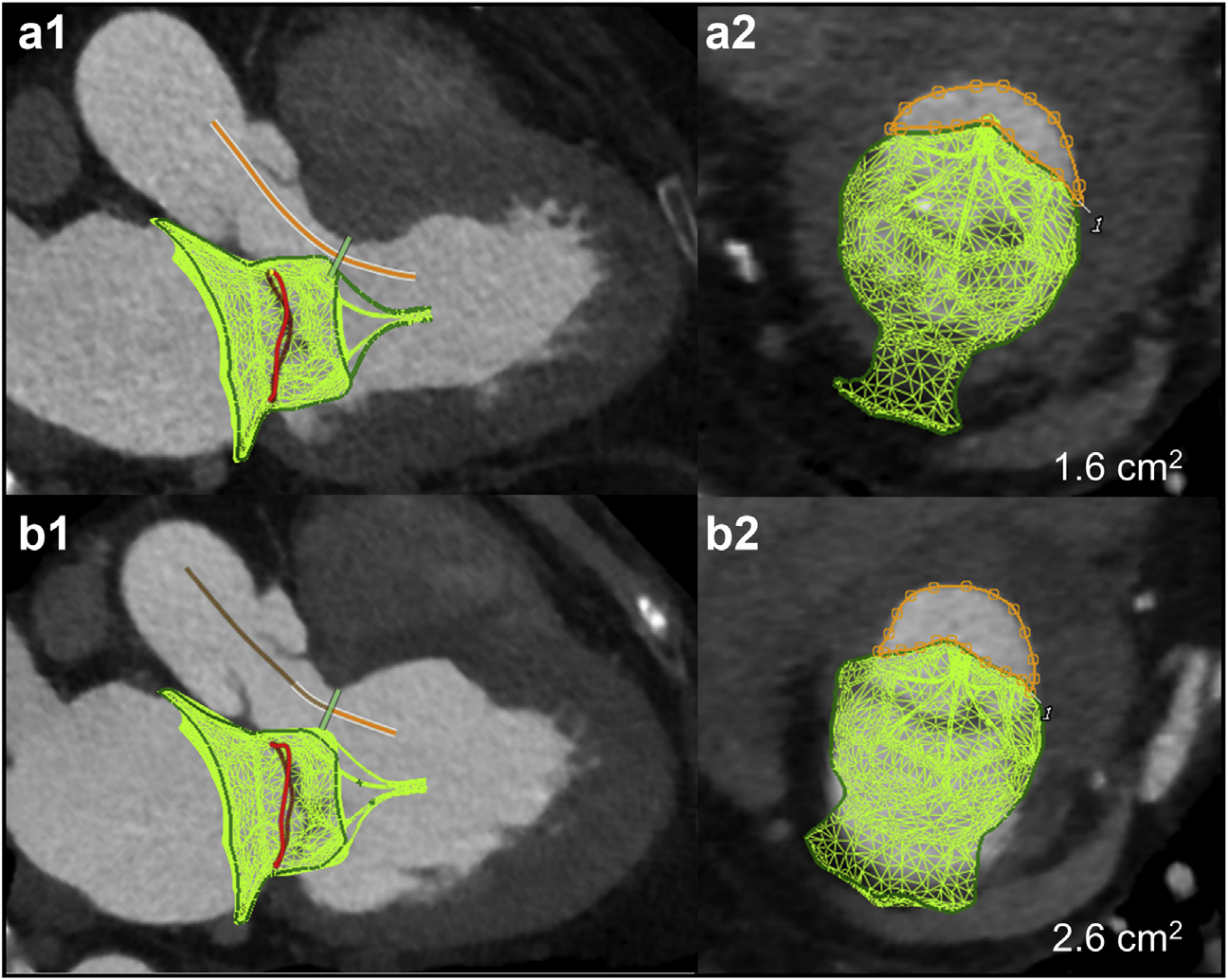
**TMVR Neo-LVOT area valve simulation with a Tendyne device modeled before and after alcohol septal ablation.** (a1 and a2) Measured neo-LVOT area before alcohol septal ablation was 1.6 cm^2^ (orange area) at midsystolic cardiac phase. (b1 and b2). MDCT scan was repeated 3 mo after pre-emptive alcohol septal ablation. Same midsystolic phase chosen with an increased neo-LVOT area to 2.6 cm^2^.

Modifications of neo-LVOT area might be necessary to reduce the risk of LVOTO post-TMVR. Techniques such as pre-emptive alcohol septal ablation (Figure 6)^12^^,^^13^ or laceration of the AML to prevent LVOT obstruction (LAMPOON) are options; however, these are limited to a few operators with technical expertise.^14^

## TMVR Access Planning

Transseptal and transapical are the 2 main access routes used for TMVR. The location of the transseptal puncture or transapical access is key to achieving optimal coaxial TMVR device deployment. Cardiac MDCT can help identify the optimal access location to achieve the perpendicular coaxial trajectory to the mitral annulus by aligning fluoroscopic projections with key anatomical landmarks.^15^, ^16^, ^17^, ^18^

**Figure 7:**
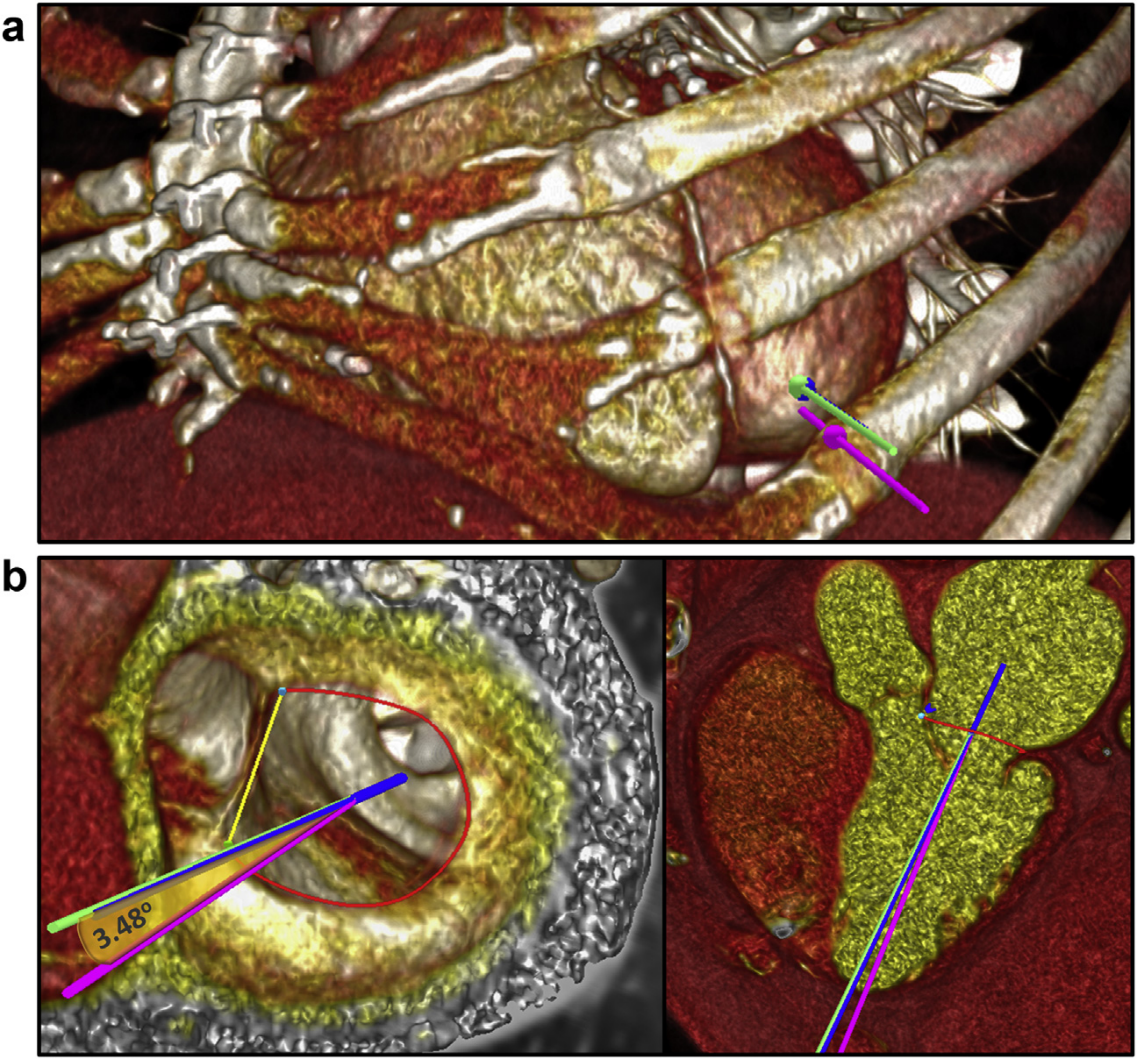
**Transapical access planning with MDCT.** (a) Volume-rendered images showing virtual handles (in pink through the true LV apex, in blue through the coaxial mitral annular trajectory, and in green the target for apical access) and their position relative to the intercostal space. In this case, as the coaxial annular handle is in the center of the intercostal space, the target (green) handle could be positioned overlapping it, which is ideal but not always possible. (b) Endoluminal volume-rendered views from the LV short and long axis showing the angle (yellow) between the target handle (green) and the true apex (pink).

For transapical access planning, once the MDCT chest data set including the heart and ribcage is loaded, a virtual handle following a straight trajectory, coaxial to the mitral annulus centroid, is simulated, and the ideal intercostal space for the transapical access is evaluated (Figure 7). Commonly, the ideal access point is located anteriorly or anterolaterally to the true LV apex, and an offset from the mitral annulus coaxial trajectory is expected.^15^ Besides the access point, careful verification of the virtual catheter trajectory related to the epicardial coronaries, papillary muscles, and chordae is essential to anticipate complications.^17^

**Figure 8:**
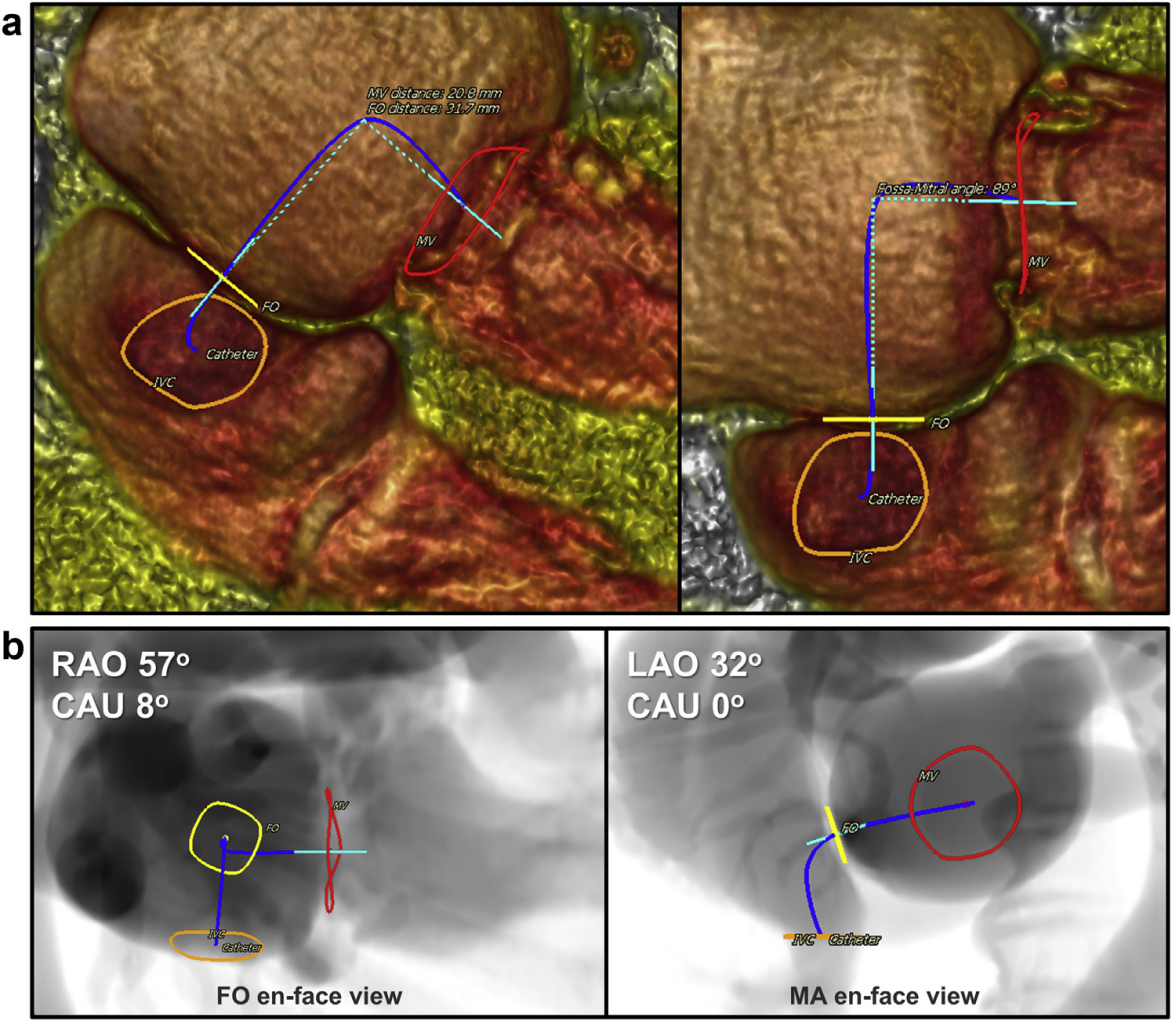
**Transseptal access planning and catheter simulation by MDCT.** (a) To help in the transseptal procedural planning, the catheter trajectory (blue) is simulated in the same mid-late systolic phase used for mitral annulus measurement. Using volume-rendered MDCT images, the simulated catheter starts from the inferior vena cava, crosses the fossa ovalis (FO), and gets to the mitral valve (MV) annulus level. Two coaxial lines, one from the center of the FO and the other from the center of the MV, are automatically drawn and connected (dashed line in light blue). The distances from the puncture site to the mitral valve and the angle to achieve the most coaxial orientation for THV deployment are estimated. (b) Fluoroscopic projections derived from MDCT for the FO en-face plane and mitral annulus (MA) en-face plane are provided to guide TEE septal puncture and THV coaxial deployment.

Cardiac MDCT can also assist in the planning of the transseptal approach. Commonly, this site is inferoposterior to achieve the best height and coaxial approach to the MV and annulus. Fluoroscopic views should be provided to help intraprocedural guidance by TEE and fluoroscopy (Figure 8). The distance from the transseptal puncture to the mitral annulus and the angle for optimal coaxial approach, as well as structural abnormalities of the interatrial septum (patent foramen ovale, atrial septal defect, septal aneurysm, and lipomatous hypertrophy), should be measured and characterized to anticipate septostomy and potential catheter navigation difficulties.^16^^,^^18^

**Figure 9:**
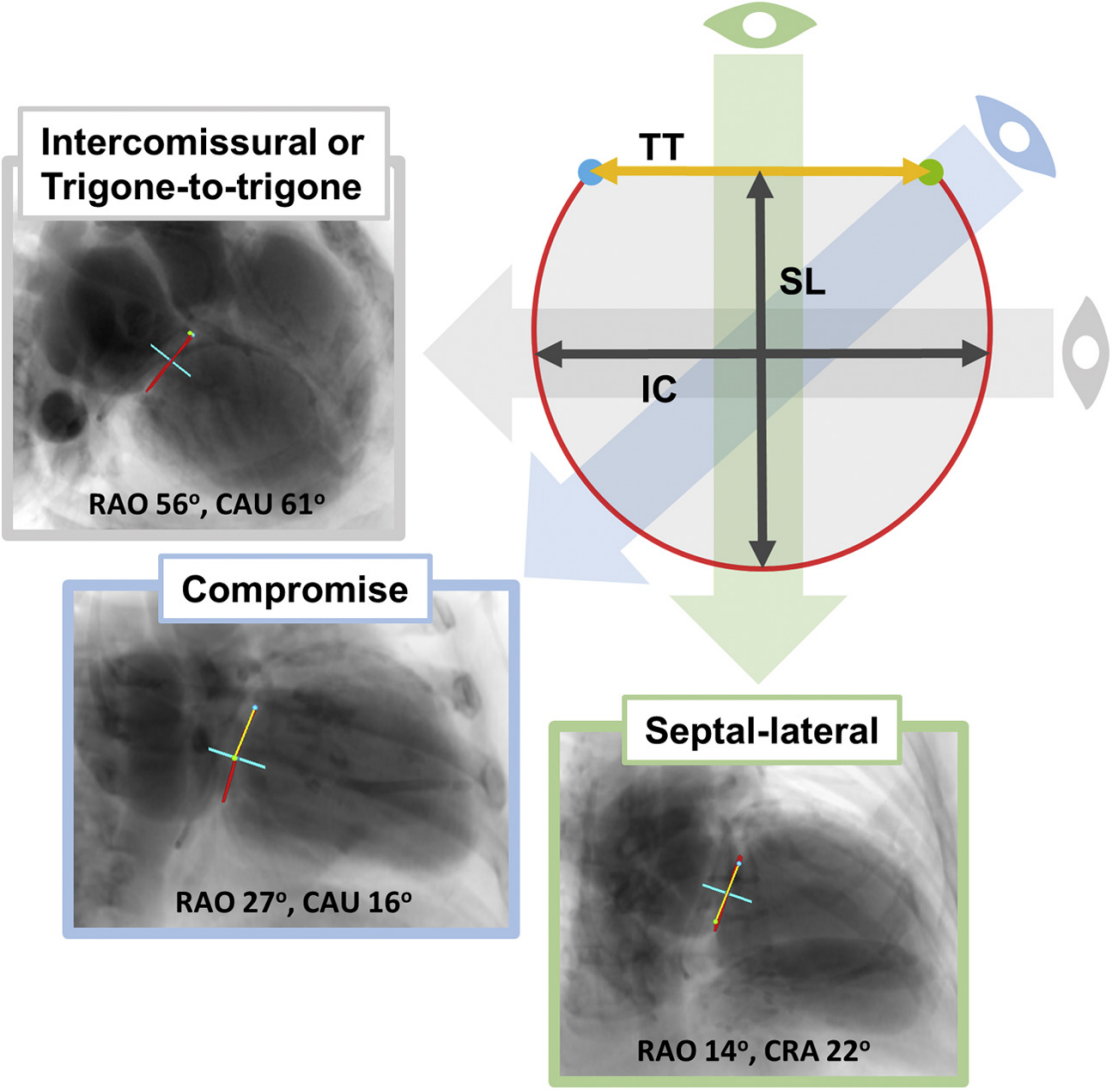
**Fluoroscopic projections assessment by MDCT.** The 3 main MDCT-derived coplanar fluoroscopic projections used for THV navigation and deployment are provided. They are named based on the directions of mitral annular measurements already described: septal-lateral (SL), trigone-to-trigone (TT), or intercommisural (IC). A compromise view between IC and SL views is usually provided due to C-arm angulation constraints to achieve an IC or TT view.

Defining the fluoroscopic angles for optimal device deployment and anchoring is also an important role of preprocedural MDCT. The septal-lateral (SL) and the trigone-to-trigone (TT) angles are orthogonal to the mitral annulus axis. The TT is in a direction parallel to the TT distance, whereas the SL is perpendicular to the TT distance, as demonstrated in Figure 9. Because of physical constraints of the C-arm and patient positioning, the SL angle may not be feasible, and a compromise view between the TT and SL angles should also be provided.^18^ Significant patient variability of these angles reinforces the role of individual planning to facilitate the procedure and optimize outcomes. This information is critical to guide the procedure in nonradiopaque degenerated bioprosthetic valves, particularly for patients receiving ViV TMVR. In addition to TEE imaging, when there is a need for additional mitral annulus reference for native TMVR, a guidewire can be inserted in the coronary sinus; using preprocedural MDCT, a virtual wire can be simulated, and the distances from the wire to the mitral annulus provided to optimize device positioning and deployment.

## TMVR for ViV

Transcatheter mitral ViV replacement is an FDA–approved TMVR treatment option for high-risk patients with degenerated surgical bioprosthetic valves. The safety and outcomes of this procedure have progressively improved, driven in large part by preprocedural MDCT planning. Recent registry data of 1529 mitral ViV TMVR procedures using the balloon-expandable THV SAPIEN 3 device had a 5.4% mortality at 30 days and 16.7% at 1 ​year, both rates substantially lower than predicted by Society of Thoracic Surgeons risk of operative mortality score.^19^

One of the first steps is to confirm, or at times to correct, the operative surgical report by identifying the size and type of the surgical heart valve (SHV).

**Figure 10:**
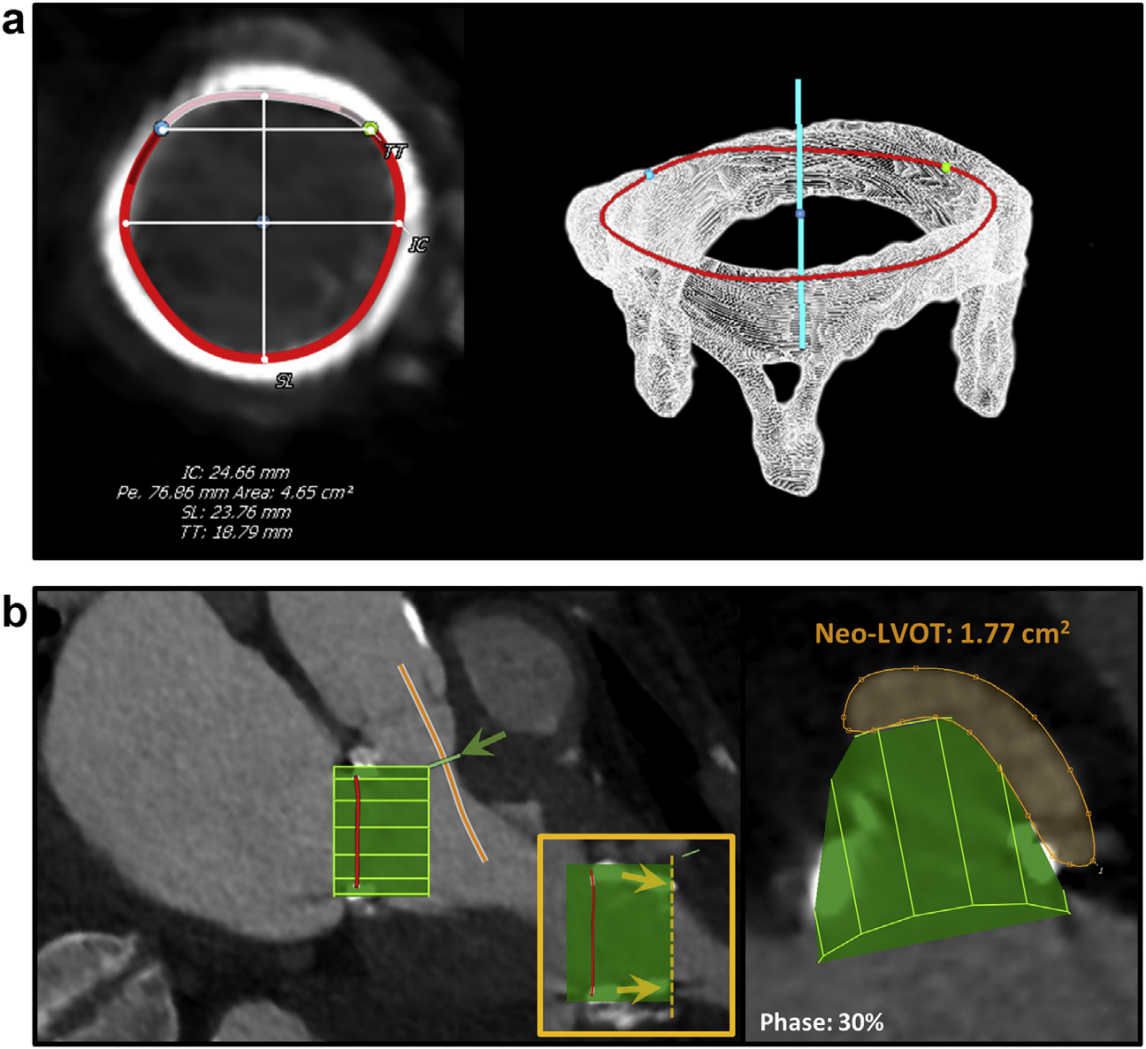
**Valve-in-valve CT planning.** (a) The internal dimensions of the degenerated SHV bioprosthesis are measured for optimal THV sizing. On the right side image, the maximal intensity projection demonstrates it to be a #25 ​mm Edwards Magna 3300 (pericardial leaflets). (b) A virtual cylinder with the same specifications provided by the manufacturer regarding width and height of a SAPIEN 3 is simulated and positioned inside the bioprosthesis. The ventricular end of the pre-existent stent posts is used as a limiting reference for the virtual THV (yellow box); if the stent posts are not radiopaque, calcification and hypodense stent posts on contrast CT images can be used as surrogates for proper positioning. Then, in the mid-late systolic phase, a centerline is traced between the virtual THV and the neo-LVOT (orange line), and a perpendicular plane is positioned at the narrowest level (green arrow). This plane, a short-axis plane represented in the last picture, is used for the Neo-LVOT area measurement as demonstrated in orange.

**Figure 11:**
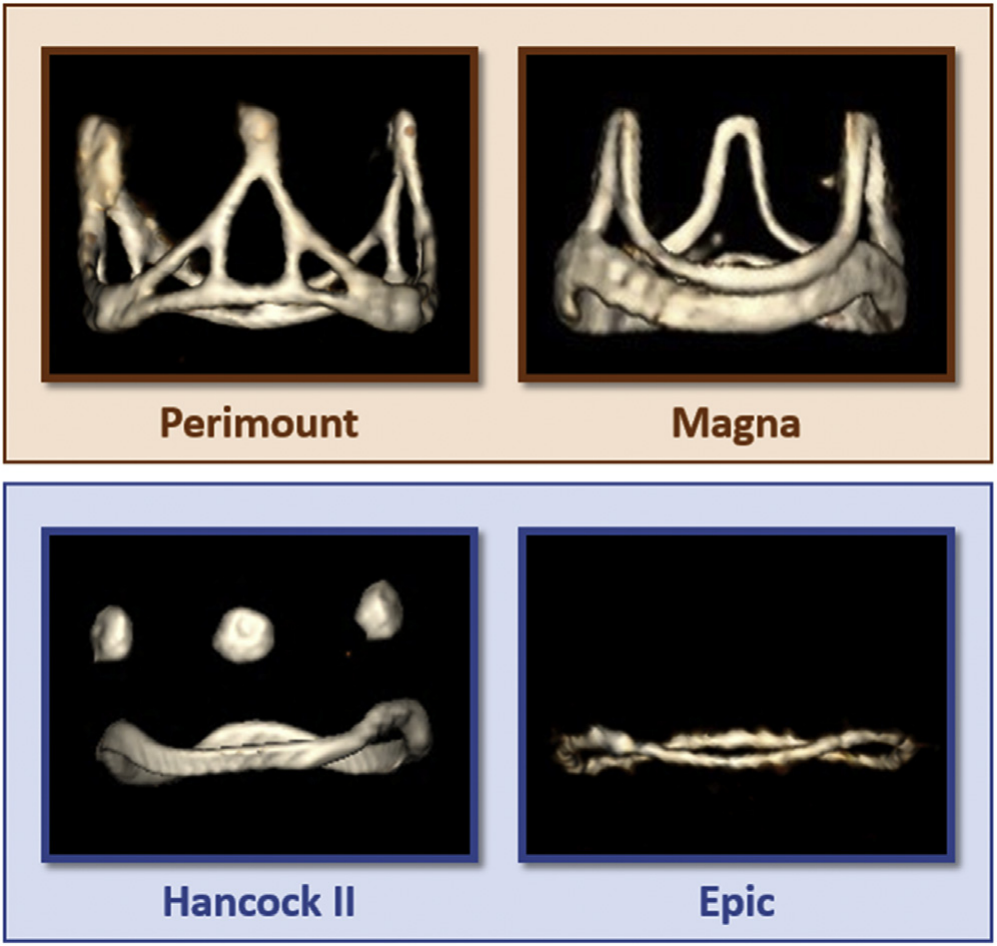
**Types and characteristics of mitral surgical heart valves by MDCT (3D maximal intensity projection rendering).** On top (orange), bovine valves; on the bottom (blue), porcine valves. Observe that the stent posts and annulus rings of bovine surgical valves are radiopaque and well defined by CT. The porcine Hancock II has 3 circular radiopaque markers on top of the stent post, but the Epic does not have a radiopaque stent post, just a thin radiopaque annulus ring.

Proper sizing of the THV for TMVR ViV procedures is critical. Oversizing is often more aggressive in mitral ViV than aortic ViV to prevent device migration and embolization, which are more prone to occur in mitral ViV due because of higher systolic closing pressure. Currently, THV sizing is mainly based on the true inner diameter (true ID) of the SHV proposed by Bapat et al., whose ViV app is a critical planning tool for SHV reference and TMVR planning.^20^ The true ID (which differs from the manufacturers’ SHV inner diameter) is the diameter accounting for the leaflet tissue sutured to the SHV stent and is at least 1 ​mm (for bovine SHV) or 2 ​mm (for porcine SHV) smaller.^21^ In cases where the SHV type is unknown or the true ID is between 2 THV label sizes (borderline true ID), direct measurement of the SHV dimensions on MDCT images using the center of the stent frame after appropriate windowing might be necessary (Figure 10). The key is to avoid extreme THV oversizing, which can cause abnormal leaflet mobility, higher gradient, and faster THV degeneration. Regarding the type and manufacturer of the surgical prosthesis, we recommend using 3D maximal intensity projections to verify the salient anatomical features of the SHV bioprosthesis (Figure 11) and confirm it according to the ViV app.

Particularly in the mitral ViV field, SHV leaflets must be considered for the neo-LVOT assessment. Porcine SHV leaflets have a shorter length (i.e., leaflets do not extend to the edge of the SHV posts) than bovine leaflets; therefore, after THV deployment, this shorter leaflet length does not cover all the THV cells interposing the LVOT, and usually, a larger neo-LVOT can be expected.^21^^,^^22^ In addition, when a balloon-expandable prosthesis is used, the left ventricular end of the device contains an open cell to maintain blood flow, which may reduce the risk of LVOTO.

The next step in the TMVR ViV planning requires simulation of the balloon-expandable transcatheter THV using specific postprocessing software. A virtual cylinder simulating the THV with adjustable diameter, height, sealing skirt height, and tilting angle is embedded in functional MDCT images and aligned with the SHV frame. The neo-LVOT area is traditionally measured in mid to late-systole with the cylinder in place, as demonstrated in Figure 10b.^23^

Factors such as THV canting, tilting, and flaring toward the LVOT may reduce the neo-LVOT area. These factors are mainly procedure-related and more difficult to simulate, but careful MDCT analysis can provide some predictors. For example, if the aortomitral angle (angle between the SHV and the aortic annulus) is obtuse (≤115°), the risk of LVOTO may increase as the THV would be oriented by the SHV struts toward the LVOT.^9^ Also, bulky and protruding posterior subvalvular calcification may push the THV more anteriorly, causing the device to cant toward the LVOT.

## TMVR for ViR

At least 25 different types of surgical mitral annuloplasty rings are currently available, with variable shapes, sizes, rigidity, and radiopacity. TMVR for ViR procedures was recently FDA-approved for high-surgical-risk patients using balloon-expandable Edwards SAPIEN 3 transcatheter aortic valve device. Given the circular shape of SAPIEN 3 THV device, the surgical annuloplasty ring must be or become circular to appropriately encircle the rounded THV during deployment. Circular rings up to size 34 ​mm, which can fit a SAPIEN 29 ​mm, are usually suitable for ViR. Oval rings, if deformable (semirigid) and complete, are also suitable (e.g., Sorin Memo 3D); however, as the ring circularizes and expands, the manufacturer’s ring dimensions are not reliable, and MDCT helps measure and estimate THV size. Incomplete rings or bands do not provide proper anchoring and are, usually, not suitable for ViR. Rigid rings provide proper anchoring, but oval- or D-shaped rings can deform the THV, leading to malfunction, intravalvular regurgitation, or (more commonly) PVL.

**Figure 12:**
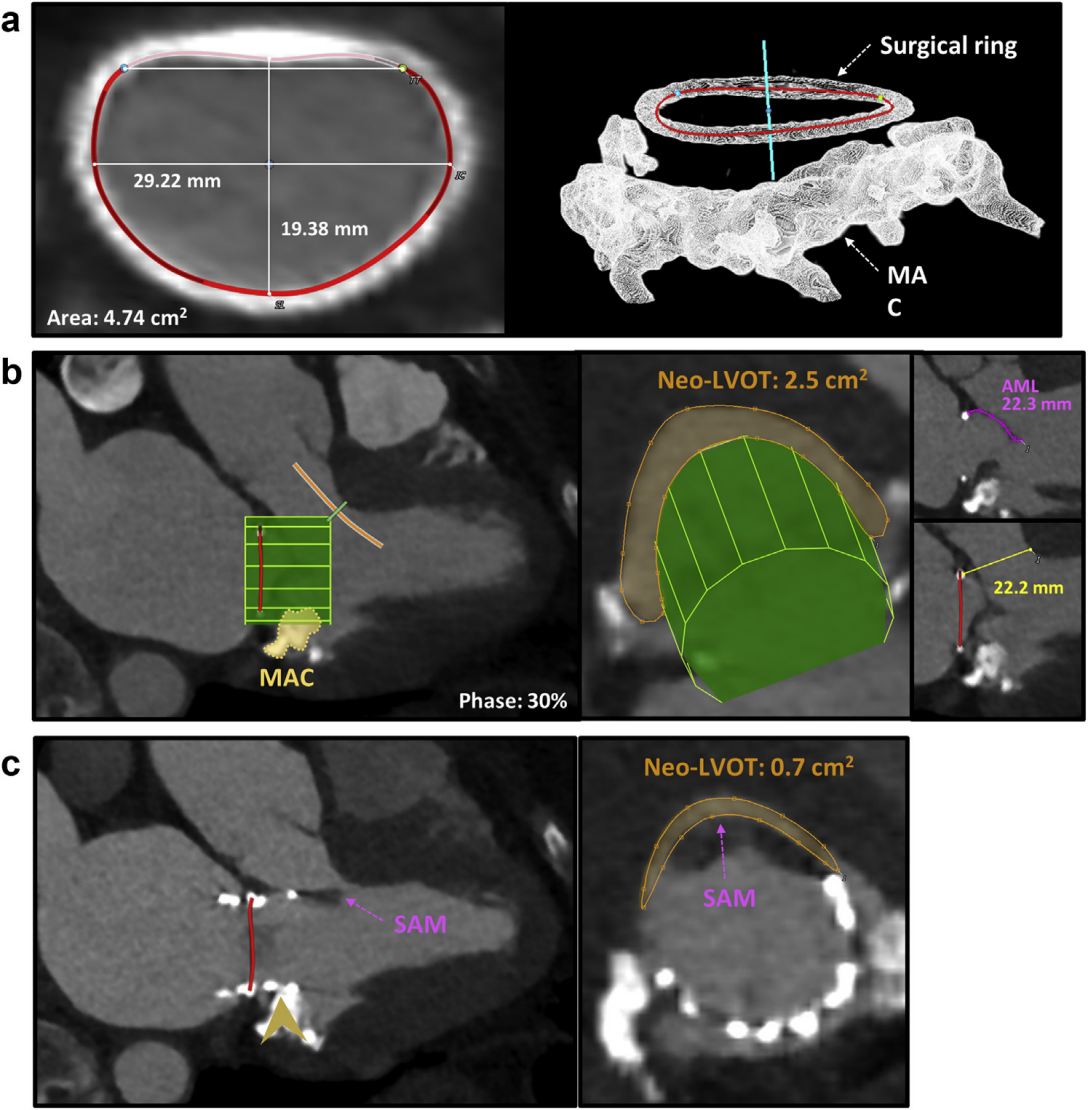
**Valve-in-ring MDCT planning and complications.** (a) Internal dimensions of a semirigid mitral annuloplasty ring (32 mm memo 3D) are measured as demonstrated. A 3D reconstruction shows, in the figure on the left, the extensive mitral annular calcification (MAC) below the ring. (b) As described, a virtual THV is positioned, and the Neo-LVOT is measured at the narrowest distance between the septum and the virtual THV. In this case, 2 factors are potential predictors of LVOT obstruction beyond the Neo-LVOT area, which in theory would be adequate (2.5 cm^2^). First, the MAC below the annulus may induce THV device canting toward the LVOT and reduce the neo-LVOT area. Second, the anterior mitral leaflet (AML) is preserved, and after device deployment, it may induce dynamic LVOT obstruction because the THV frame will dislocate it anteriorly toward the LVOT. The anterior mitral leaflet has the same length as the ring-to-septal distance, indicating a higher risk for this complication. (c) In the same case, a post valve-in-ring MDCT has shown the 2 potential complications described in panel b. The posterior MAC induced device canting (yellow arrowhead) toward the LVOT, and most importantly, a systolic anterior motion (SAM) of the AML caused systolic LVOT obstruction.

The neo-LVOT assessment for ViR procedures is similar to ViV and ViMAC and is described in Figure 12. Previous data have shown higher rates of LVOTO in ViR compared with ViV procedures.^9^^,^^24^ In contrast with surgical MV replacement, the AML is often preserved after surgical mitral annuloplasty and can lead to LVOTO even with neo-LVOT area >1.9 ​cm^2^. After ViR TMVR, the AML is displaced anteriorly (toward the septum) by the THV stent frame. When the AML is elongated, it may extend beyond the ventricular edge of the THV. This overhanging and often mobile part of the AML may move anteriorly toward the interventricular septum during ventricular systole leading to dynamic LVOTO^25^ (Figure 12b) or retroflex back into the new valve, causing an obstruction. The combination of an elongated AML (i.e., >25 mm in length) with a small, hypertrophic, and hyperdynamic left ventricle and a short distance from the mitral annulus to the interventricular septum increases the risk for LVOTO in patients with borderline neo-LVOT area.^9^^,^^25^ For these patients, suitability for pre-emptive alcohol septal ablation or transcatheter longitudinal splitting of the AML (LAMPOON procedure) before TMVR ViR should be considered.^13^^,^^14^

## TMVR for ViMAC

ViMAC using balloon-expandable THV or specific TMVR devices are treatment options for high-surgical-risk patients. Cardiac MDCT has been an integral component for preprocedural ViMAC planning by providing (1) anchoring parameters (MAC distribution and characteristics), (2) landing zone and oversizing dimensions, (3) neo-LVOT area and other predictors of LVOTO.^23^^,^^26^

MAC is a subannular process where the THV device fixation will occur. The MAC circumferential extent (in degrees) and its characteristics (thickness, density, and irregularities) are important predictors of THV migration or embolization, especially for devices in which the anchoring mechanism is through radial force (e.g., balloon-expandable SAPIEN 3 THV). Circumferential and contiguous calcification that involves the fibrous trigones, preferentially not brittle or spiked, is required for proper THV anchoring. A recent MDCT-derived score was developed to predict THV migration or embolization for balloon-expandable THV using a combination of 4 MAC features^27^: calcium thickness (<5 mm = score 1; 5-9.99 mm = score 2; ≥10 mm = score 3), degrees of annulus involvement (<180° = score 1; 180°-270° = score 2; ≥270° = score 3), fibrous trigones calcification (one trigone = score 1; both trigones = score 2), and leaflets calcification (1 leaflet = score 1; 2 leaflets = score 2). When the MAC score was 6 or less, the rate of migration and/or embolization was 60%; when 7 or greater, the rate was only 9.7%. In addition to the MAC score, adequate oversizing is key to avoid THV migration or embolization. The calcium density and the presence of protruding calcium spicules may increase the risk of irregular THV device expansion, leading to premature dysfunction and PVL.

**Figure 13:**
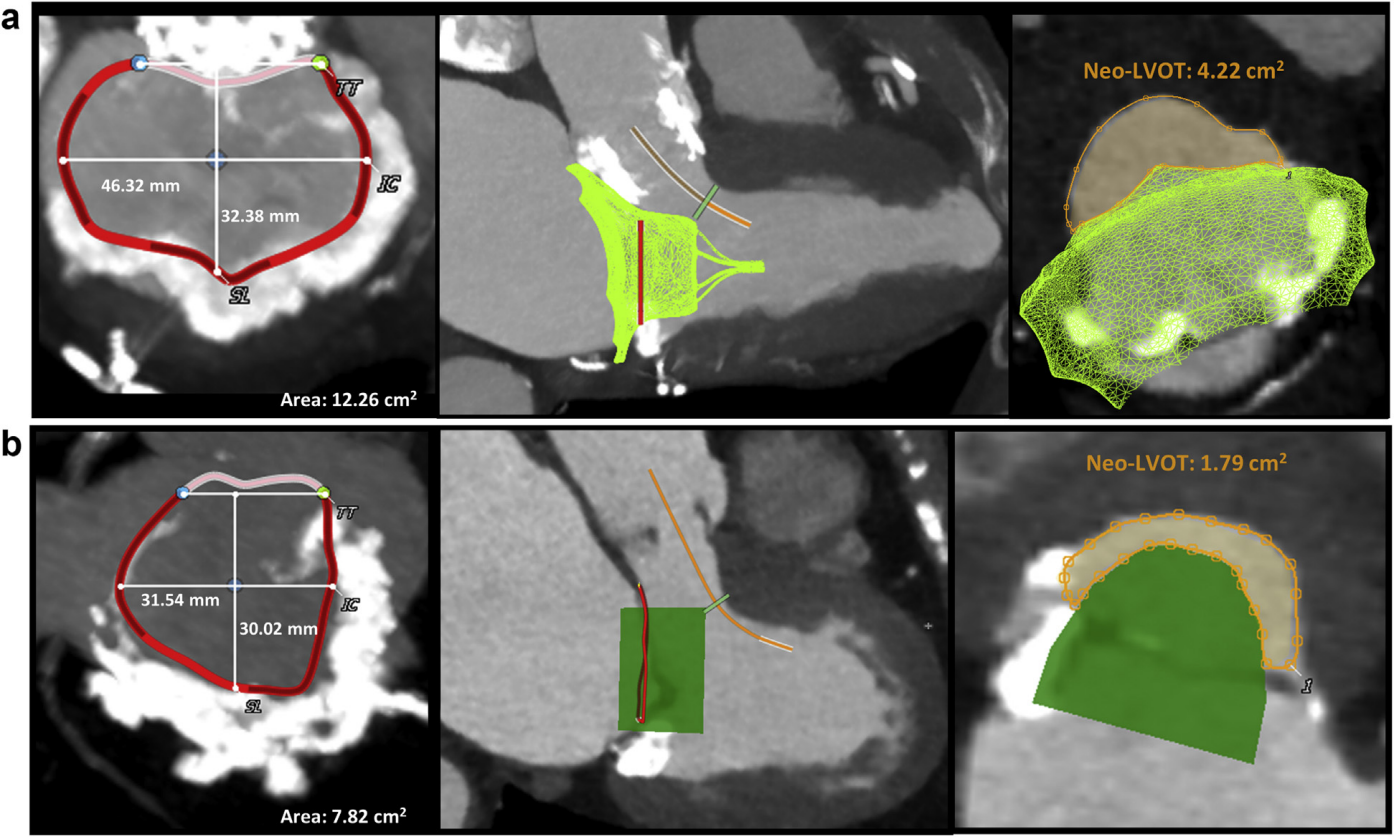
**TMVR and valve-in-MAC MDCT planning.** Case examples are showing a Tendyne-in-MAC (a) and a valve-in-MAC (b) simulation. Internal MAC dimensions for valve sizing using the D-shaped methodology previously described and the neo-LVOT area measurement for each case are demonstrated.

The MAC contour is irregular and has a 3-dimensional distribution, making accurate annulus or landing zone sizing more variable. Although the mitral annulus measurement methodology is not standardized for these patients, a reasonable approach is using a multiplanar reconstruction with a thick slab and maximum intensity projections.^28^ The narrowest short-axis (en-face) plane, including the entire MAC, is reformatted, and its inner border is traced using a spline tool and D-shaped annulus truncation. A smooth inner tracing, including the irregular edges and protruding spicules, is preferred (Figure 13).

The rates of LVOTO after ViMAC are the highest of the TMVR procedures, and the assessment of MDCT predictors is key.^9^ The neo-LVOT is often measured using a similar simulation methodology to other TMVR procedures (Figure 13) and is the main predictor of LVOTO; however, other significant factors should be considered. In contrast with ViV and ViR where the THV expansion toward the LVOT is restricted by the annuloplasty or the sewing ring, in ViMAC, the AML and fibrous trigone calcification anchor the THV. Because of their variable compliance, the neo-LVOT area and LVOTO are less predictable even in the presence of an area >1.7 cm^2^. A higher cutoff may be used for ViMAC procedures to account for this factor. Similar to ViR, the presence of the AML may cause a dynamic LVOTO because of systolic anterior motion, and its length should be measured as already described (Figure 12b).

The Tendyne TMVR device (Abbott Structural Heart, Menlo Park, California) is under investigation for use in symptomatic patients with MR (with or without MAC) through the SUMMIT trial (NCT03433274). Its unique design and anchoring mechanisms offer potential advantages compared with balloon-expandable THVs. Initial multicentric data demonstrated Tendyne’s safety and effectiveness.^6^ Its D-shaped outer stent with the straight edge abutting the AML follows the native valve shape and reduces the expansion toward the LVOT. Also, its unique anchoring mechanism (a polyethylene tether connected to an apical epicardial pad) reduces the dependency of radial anchoring and overexpansion of the mitral annulus.^6^^,^^29^

## Post-TMVR Imaging Assessment

The echocardiographic assessment post-TMVR evaluates LV function, device stability, LVOT velocity, residual MR or PVL, and postprocedural MV gradients. Assessment of new TMVR device leaflet mobility, presence/absence of hypoattenuated leaflet thickening, and device malposition can be challenging to assess by transthoracic echocardiography. Although routine post-TMVR MDCT imaging surveillance is not recommended outside of research protocols, post-TMVR MDCT should be considered whenever echocardiographic imaging is suboptimal, findings are ambiguous, and especially when transmitral gradients are elevated (>5-6 mmHg).

**Figure 14:**
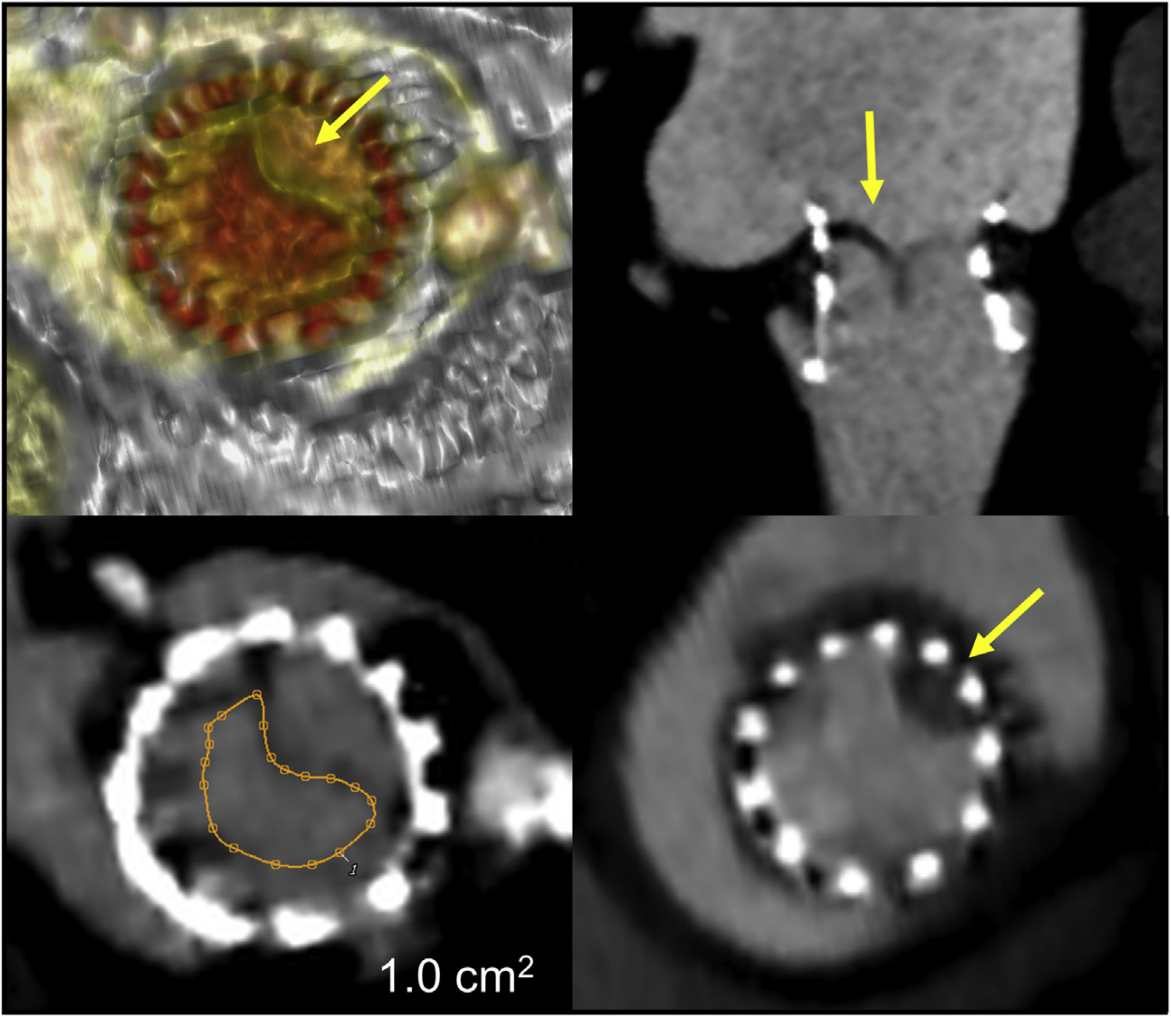
**Evaluation of prosthesis leaflet mobility after transcatheter mitral valve replacement by MDCT.** A patient who received ViV TMVR presented with elevated transmitral gradient of 7 mm ​Hg seen on 1 mo post-TMVR echocardiography study. Hypoattenuating leaflet thickening (HALT) with restricted mobility of the 1 leaflet (yellow arrow) is seen on functional MDCT. The THV opening area is as small as 1.0 cm^2^ (orange tracing).

The post-TMVR MDCT imaging acquisition protocol is similar to pre-TMVR implant with full cardiac cycle coverage and optional dose modulation. Post-TMVR hypoattenuated leaflet thickening with or without restricted leaflet mobility can be detected by MDCT even the mean transvalvular pressure gradient is minimally elevated (Figure 14). Post-TMVR device geometry can be clearly defined on post-TMVR MDCT. Given the necessary oversizing for TMVR device fixation, compression causes stent frame deformation and change of device shape after implant, making the predicted LVOT risk and actual postimplant LVOT area measurements different.^11^ Evaluation of LV remodeling after TMVR is also important and can be evaluated by MDCT depending on the site of the apical pad with Tendyne for preferred LV remodeling.^30^ For additional device implant post-TMVR (e.g., left atrial appendage closure devices), the possibility of mutual interference between devices should be assessed by MDCT.^31^

## Conclusion

Cardiac MDCT is a fundamental diagnostic imaging tool for TMVR planning, enabling comprehensive evaluation of patient cardiac anatomy and function, which informs patient suitability, device selection, and risk of potential complications. The knowledge continues to evolve in this dynamic field, and constant developments require training and immersion from imagers involved to gain expertise in the planning of these procedures.

## Funding

The authors have no funding to report.

## Disclosure statement

J.L.C. has received consulting fees from Abbott Vascular, Aria CV, Boston Scientific, Edwards Lifesciences, Medtronic, Gore WL, TriFlo, Xylocorand; has received institutional research grants from 10.13039/100006520Edwards Lifesciences, 10.13039/100008497Boston Scientific, 10.13039/100011949Abbott Vascular; and 10.13039/100016262Abbott Northwestern Foundation; research support from Circle Cardiovascular Imaging, 3Mensio, and 10.13039/501100011699Siemens Healthineers. P.S. has received consulting fees from Abbott Structural, Medtronic, Boston Scientific, Edwards Lifesciences Admedus, Gore and Teleflex; has received research grant support from Abbott Structural, 10.13039/100004374Medtronic, and 10.13039/100008497Boston Scientific; and has been a speaker for Abbott Structural. S.G. is a consultant for Medtronic, Edwards Lifesciences, and Abbott Vascular; received institutional research grants from 10.13039/100006520Edwards Lifesciences, 10.13039/100011949Abbott Vascular, Gore, and 10.13039/100008497Boston Scientific; and is a proctor for Edwards Lifesciences. M.G. has received consulting fees from Abbott Vascular and Edwards Lifesciences. All other authors have reported that they have no relationships relevant to the contents of this paper to disclose.
